# Analysis of Wnt signalling dynamics during colon crypt development in 3D culture

**DOI:** 10.1038/srep11036

**Published:** 2015-06-18

**Authors:** Chin Wee Tan, Yumiko Hirokawa, Antony W. Burgess

**Affiliations:** 1 Structural Biology Division, The Walter and Eliza Hall Institute of Medical Research, 1G Royal Parade, Parkville, VIC 3052 Australia; 2 Department of Medical Biology, University of Melbourne, 1G Royal Parade, Parkville, VIC 3052 Australia; 3 Department of Surgery, University of Melbourne, Royal Melbourne Hospital, Parkville, VIC 3050, Australia

## Abstract

Many systems biology studies lack context-relevant data and as a consequence the predictive capabilities can be limited in developing targeted cancer therapeutics. Production of colon crypt *in vitro* is ideal for studying colon systems biology. This report presents the first production of, to our knowledge, physiologically-shaped, functional colon crypts *in vitro* (i.e. single crypts with cells expressing Mucin 2 and Chromogranin A). Time-lapsed monitoring of crypt formation revealed an increased frequency of single-crypt formation in the absence of noggin. Using quantitative 3D immunofluorescence of β-catenin and E-cadherin, spatial-temporal dynamics of these proteins in normal colon crypt cells stimulated with Wnt3A or inhibited by cycloheximide has been measured. Colon adenoma cultures established from APC^min/+^ mouse have developmental differences and β-catenin spatial localization compared to normal crypts. Quantitative data describing the effects of signalling pathways and proteins dynamics for both normal and adenomatous colon crypts is now within reach to inform a systems approach to colon crypt biology.

Colorectal cancer (CRC) is one of the most common forms of cancer and a leading cause of death in the western world^1^. CRC initiation arises when there is a loss of control over proliferation and migration of colon crypt cells^2^,^3^. It has been reported that cell proliferation and crypt production processes have critical roles in the growth of colorectal adenomas and hyperplastic polyps^4^. Subsequent reports further implicate the Wnt signalling pathway in intestinal crypt formation, cell proliferation^5^,^6^,^7^ and the regulation of cell-cell adhesion in crypts^8^,^9^. It is therefore inevitable that the Wnt/β-catenin signalling pathway has attracted considerable attention with 90% of CRCs having mutations in key components of this pathway, i.e. adenomatous polyposis coli (APC) or β-catenin (β-cat)^10^.

Currently, the processes of crypt formation and the roles of Wnt signalling or cell-cell adhesion are not well described in the colon. Similarly, the links between the underlying APC, β-cat and E-cadherin (E-cad) biochemistry and adenoma formation are still unclear. To improve the development of targeted therapeutics for CRC, a quantitative understanding of the molecular and cellular events leading to aberrant colon crypt development (due to APC mutation) and the subsequent effects of oncogene mutation on the characteristics of CRC stem cells is essential^11^.

A computational systems biology approach^12^ is required to assess the non-intuitive behaviours of Wnt signalling and will provide an integrative perspective of both normal crypt production and cancer progression^13^. However, critical cell-level, tissue-level and disease-relevant quantitative data (spatial and dynamic responses of signalling proteins to bioactive stimuli) essential for producing robust models capable of predicting reliably crypt development, the effects of perturbation of tumour suppressor genes, the activation of proto-oncogenes or crypt responses to anti-cancer therapeutics^14^,^15^ are lacking.

Historically, drug development has relied on *in vitro* experimentation of cancer cell lines. Often these cell line fail to translate into accurate predictions of patient responses to anti-cancer treatments^16^,^17^. The improvements in colon crypt culture and analysis described in this report create new opportunities for investigating tissue morphogenesis and the effects of anti-cancer drugs for CRC.

Previous studies on intestinal cell biology have primarily focused on small intestine cultures^18^,^19^. It is only recently that colon epithelial cultures^20^,^21^,^22^,^23^ became a plausible experimental model for studying *in vitro* colon biology and CRC. We have recently established a colon crypt co-culture system using a colon sub-epithelial myofibroblast cell line as a support cell^23^. In culture, isolated colon crypts round up to form multicellular spheres (colonospheres), with many progressing on to form colonoids (colonospheres with multiple crypt-like structures). This study focuses on developing a context-specific culture system for studying colon epithelium. We optimized the culture setup to allow time-lapsed imaging to monitor morphological changes from the initial crypt (or fragment) to colonosphere to colonoid for up to 10 days. Surprisingly, two distinct mechanisms of *in vitro* crypt formation were revealed: 1) a crypt formation event producing a single crypt derived from a colonosphere early in the cultures and; 2) crypt formation events emerging from large colonospheres later in the cultures. The *in vitro* crypts formed using this optimized setup had morphologies which were similar to that of colon crypts *in vivo*. The setup was further used to grow colon adenoma derived from APC^min/+^ mouse. The ability to grow these colon crypts in culture provides opportunities for “normal vs adenomatous” colon-specific cell biology and protein distribution data to be acquired. We have previously generated quantitative data on Wnt signalling proteins in cultured cells relevant to human CRC^24^ using quantitative western blots and 3D confocal immunofluorescence microscopy, obtained protein dynamics data of β-cat in human kidney cells^25^ as well as measured distribution of β-cat and E-cad in colon crypts isolated from mice^26^. By combining these techniques, we measured Wnt signalling protein dynamics during the formation of colon crypts in culture.

## Results

### Simplification of the colon crypt culture system improves the quality of colonoids

**Figure 1:**
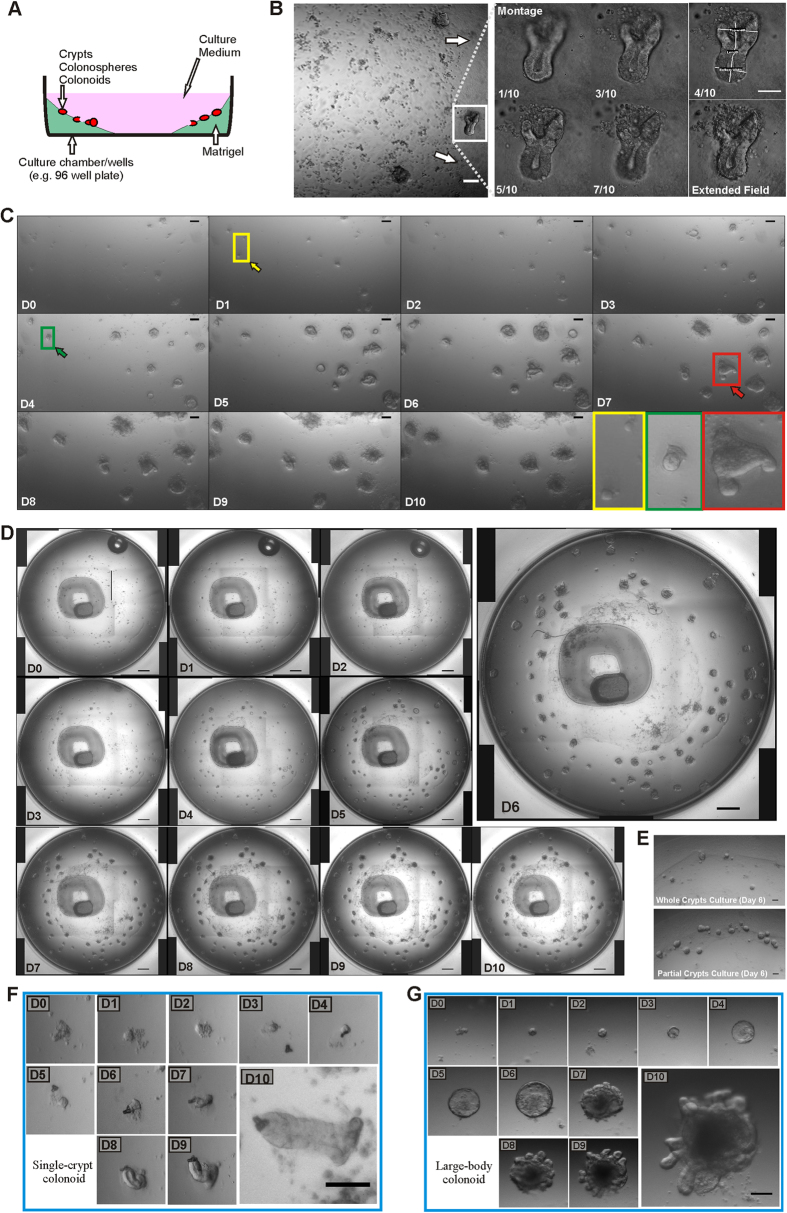
Optimized colon crypt culture setup produces single-crypt colonoids. (**A**) Culture setup: A layer of Matrigel was laid along the circumference of the well/chamber with the crypts suspension plated over the top of the Matrigel and allowed to settle. (**B**) Single-crypt colonoid (boxed): 2D image of day 3 colon crypt culture with the Matrigel gradient increasing towards the wall (arrows). 2D Montage bright field images at different focal planes with annotations (top, base, width and length) and the 2D-‘Extended Depth of Field’ image generated from 10 confocal images. (**C**) 2D-EDOF images of colon crypt cultures from day 0 to 10 showing the rounding up of crypt fragments into colonospheres in the first 2 hours (yellow box), forming crypt-shaped colonoids by day 4 (green box) and large-body colonoids from day 5 onwards (red box). (**D**) Whole well 2D-EDOF monitoring of crypts/colonospheres for up to 10 days. (**E**) Bright field images of day 6 colon culture from either whole crypt (top) or partial crypt (bottom) cultures, indicating a better growth for the partial crypt cultures. (**F**–**G**) Magnified 2D-EDOF images of crypt cultures monitored for 10 days, showing the two types of *in vitro* crypt formation mechanisms: (**F**) single-crypt colonoid, (**G**) large-body colonoid. Single-Crypt colonoid reshape from a colonosphere into a crypt-shaped structure while large-body colonoid presents multiple new crypts budding off a large colonosphere-like structure. Scale bars: (**B**) 50 μm, (**C**) 100 μm, (**D**) 500 μm and (**E**–**G**) 100 μm.

Using the optimized colon culture setup (See Methods and Fig. 1A), we were able to measure colon crypt development quantitatively. Under these culture conditions, during the early stages of the culture, single colon crypts with almost physiological morphology form (Fig. 1B). These *in vitro* crypts have similar dimensions to crypts isolated from normal, adult mouse colons. Specifically, the average bottom width of these crypt-shaped structures is 50.6 ± 10 μm (n = 8), similar to the 43.5 ± 6.1 μm (n = 237) for crypts isolated from the distal colon of 7 week old mice^26^. This optimized colon culture system facilitates the rounding up of crypt fragments within the first 24 hours, the formation of single crypts by day 4 and supports colonoid growth for up to 10 days (Fig. 1C). Single crypts are not observed in the conventional 3D cultures where the crypts or fragments are embedded in Matrigel^21^,^23^.

This colon culture setup permits the integration of 3D microscopy and image processing techniques to monitor, track and quantify crypt formation in real time. Time-lapse imaging enabled the entire culture well to be imaged daily (up till day 10) using overlapped sequenced images at multiple focal depths (Supplementary Fig. S1). Individual crypt development (from fragment to colonosphere to colonoid) was tracked using the time-lapsed information from each well (Fig. 1D). Semi-digested crypts (i.e. the crypt bottoms, see Fig. 1C–D day 0) grow more efficiently than whole crypts (Fig. 1E), corroborating Sato *et al*’s^21^ report that fragments of small intestinal crypts form spheroids more efficiently than intact crypts.

Two types of crypt formation occur in these colon cultures. Type 1: crypt formation occurs when a small colonosphere reshapes to form a single crypt (Fig. 1F); Type 2: crypts project out from a large colonosphere (diameter > 200 μm, Fig. 1G). These two types of crypt formations are term single-crypt colonoids and large-body colonoids respectively. After 10 days, the colonoids were either passaged or fixed for immunofluorescent microscopy (as both the colonoids and Matrigel start to break down after that time).

### Effects of withdrawing noggin on Colon crypt formation

**Figure 2:**
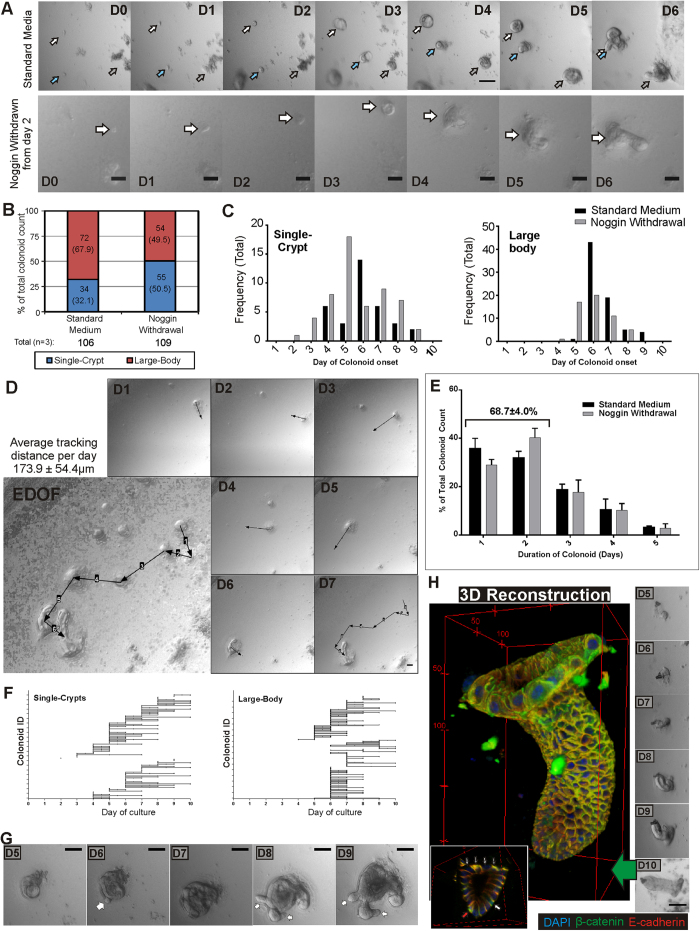
Colon crypt’s development in vitro and effect of noggin withdrawal. (**A**) Colon culture monitored and imaged for 10 days with either standard medium or standard medium with noggin withdrawn from day 2. Magnified 2D-EDOF images of these colon cultures from day 0 to day 6 shown, indicating the appearance of colonoids from day 4. (**B**) Colonoids were scored and classified (single-crypt or large-body) visually from the analysis of 2D-EDOF images (n = 3) from A. Proportion of total colonoids counted for each category (%) is shown in brackets. Noggin withdrawal led to an increase in single-crypt colonoids while total colonoid counts remained similar. (**C**) Frequency of colonoid onset (day of initial formation) for both single-crypt (left) and large-body (right) colonoids. Single-crypt colonoids appeared earlier than large-body colonoids, with noggin withdrawal leading to earlier onset of colonoid formation. (**D**) 2D-EDOF image tracking (arrow: distance travelled in next 24 hours) of a colonosphere as it forms a single-crypt colonoid by day 7, showing the highly mobile nature of the culture with an average distance of 173.9 ± 54.4 μm. (**E**) The colonoid persistence duration was tabulated for both single-crypt and large-body colonoids. 69% of colonoid events are single/two day events. (**F**) The individual colonoids durations showing their onset and persistence in culture, indicating that short-term crypt budding events commence from day 3. (**G**) 2D-EDOF images of an individual colonoid (day 5 to 9) showing a crypt bud on day 6, retracting in day 7 before re-emerging by days 8. (**H**) Immunofluorescence for β-cat and E-cad of a single-crypt colonoid (day 10, RGB 3D reconstruction) formed from a small colonosphere (see panels on right). This structure is physiologically similar in shape to crypts isolated from mice colons, with a top lumen opening and circular shaped bottom. Heterogeneity of β-cat and E-cad expression was observed at the crypt base as seen from the orthoslice views (inset). Scale bars: (**A**, **G** and **H**) 100 μm, (**D**) 50 μm.

This culture setup allows biochemical perturbations to be studied. We tested the effects of the withdrawal of noggin from the culture medium from day 2 (Fig. 2A). Single-crypt colonoids form in the absence of noggin but at different frequency. Quantitative analysis of the two types of colonoids in standard cultures showed single-crypt colonoids constitute 32% of total colonoids formed during the 10 days (Fig. 2B). When noggin was withdrawn at Day 2, the frequency of single crypt colonoids increased to ~50% of total colonoids (Fig. 2B) with a corresponding decrease in the frequency of large-body colonoids (67.9% to 49.5%). Furthermore with noggin withdrawal, the first occurrence of the single-crypt colonoids is brought forward from day 4 to 2, while for the large-body colonoids, from day 6 to 5 (Fig. 2C).

Daily image analysis of the colon crypt cultures enables the measurement of crypt formation events throughout the culture. Interestingly, the colonospheres/colonoids migrate substantial distances each day (Fig. 2D, 170 ± 50 μm average daily distance). Furthermore, crypt formation is predominantly a short-term event: 69 ± 4.0% of the colonoids persists for less than 3 days as shown in Fig. 2E (histogram of the duration (persistence in days) of each crypt formation event (both single-crypt and large-body colonoids)).

Figure 2F shows the individual event durations (“life-times”) for each colonoid scored (single-crypt and large-body) during the 10 days. The majority of crypt formation life-times were short (1 or 2 days), highlighting the short-term nature of crypt formation *in vitro*. Transient crypt forming events were also observed where the crypt buds started to emerge on one day, retracting back into the colonosphere or colonoid body the following day before re-emerging the next day (Fig. 2G). The implication of this behaviour is that simply scoring for crypts numbers at particular time-points is unlikely to provide a reliable measure of the crypt forming potential in these cultures.

To verify that the single-crypt colonoids were similar in structure to crypts isolated from mouse colons, the colonoids were harvested, immuno-stained for β-cat and E-cad and imaged under a confocal microscope. 3D-image reconstructions were generated: a typical single-crypt colonoid image is shown in Fig. 2H (also Supplementary Video S1). From the 3D image, the top lumen opening and crypt base can be identified clearly. The morphology of the single-crypt colonoid derived *in vitro* was similar to freshly isolated colon crypts^26^ (Supplementary Fig. S2C) and *in situ*^5^. Furthermore, the heterogeneous expression of β-cat and E-cad that was observed in the isolated crypts^26^ can also be seen in these colonoids (Fig. 2H inset). Significantly, the single crypt colonoids are the first physiologically-shaped colon crypts, to our knowledge, to be grown *in vitro*.

### High resolution imaging of colonoids reveals heterogeneity of β-cat and E-cad of the crypt buds

**Figure 3:**
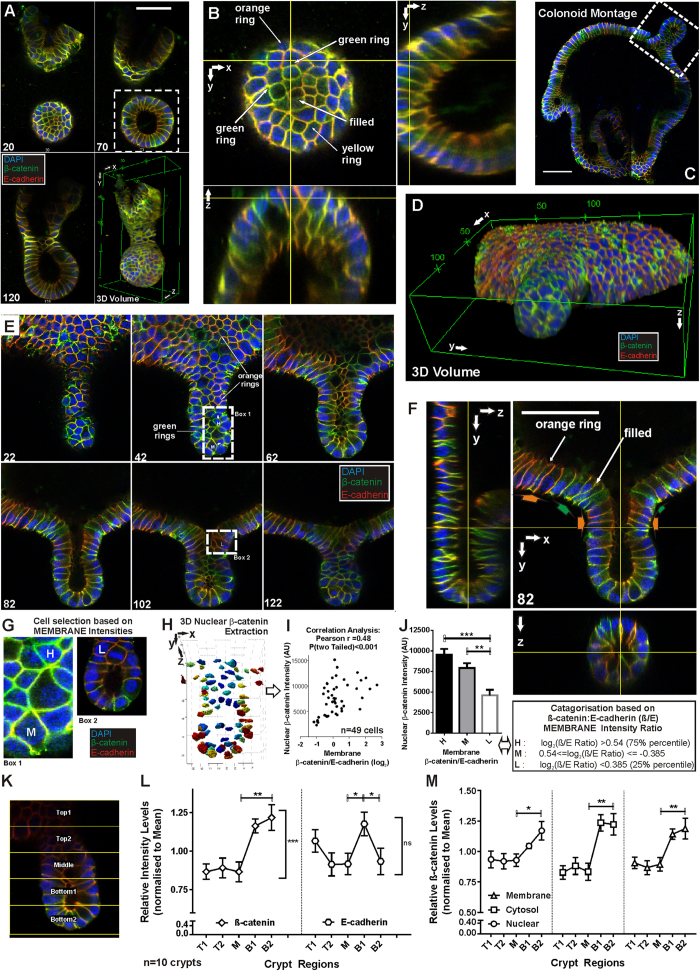
Regional distribution and heterogeneity of β-cat and E-cad in colonoid. Colon colonoids shown as immunofluorescent images extracted from image stacks (DAPI: blue, β-cat: green, E-cad: red). (**A**) 2D sections and 3D reconstruction of a day 3 single-crypt colonoid which appeared as a crypt-shaped structure. (**B**) Orthogonal views of colonoid in A showing heterogeneous expression of β-cat and E-cad as variable coloured rings at the bottom surface (dotted box in A). (**C**-**F**) Day 7, some of the colonoids had developed into structures >150 μm wide with crypt buds emerging in various directions. (**C)** Montage of confocal images showing the size of such a colonoid. (**D**) 3D reconstruction of the crypt bud in C showing heterogeneous surface β-cat and E-cad staining. (**E**) 2D sections through the depth of the bud in C showing E-cad-high (orange) cell clusters amongst E-cad low/β-cat high (green) cells. (**F**) Orthogonal views of the bud showing the well-formed structure similar to an *in vivo* crypt. (**G**) Cells with distinct membrane localization of E-cad and/or β-cat were visually selected, the intensities along their membranes boundaries (dotted) quantified and classified based on their β/E ratios (*L, M* and *H*). (**H**) The corresponding 3D nucleus for selected cells in G were matched, β-cat intensity extracted (I) tabulated with respective β/E ratios or (**J**) categories according to the β/E ratios (n =49 ± SEM, p < 0.01 one-way anova). A lower β-cat level was observed for cells with low β/E ratio. (**K**) β-cat regional compartment quantitation for *in vitro* colonoid crypts (n = 10) where each crypt’s image stack was divided into 5 equal regions. (**L**) Relative total β-cat and E-cad intensity levels along the crypt length (Top T* to middle M to bottom B*) were tabulated showing significantly higher β-cat level at the bottom (p < 0.01, M vs B2, p < 0.001 whole length) whereas the corresponding E-cad level was generally unchanged. Notably, E-cad was significantly higher in region B1 (p < 0.05, ± SEM). (**M**) In all sub-cellular compartments, β-cat intensity are higher at the bottom regions of the colonoid crypts (p < 0.05, ± SEM). Statistical test: Single factor anova. See Supplementary Video S2 for animation of a single-crypt colonoid.

Colonoids and colonospheres from day 2 were fixed and immuno-stained for β-cat and E-cad as per our previous studies on isolated colon crypts^26^ with DAPI used to mark the nucleus. A single-crypt colonoid at day 3 and a large-body colonoid at day 7 are displayed in Fig. 3. Heterogeneity of β-cat (in green) and E-cad (in red) expression can be observed with clusters of stained cells appearing as bright green (high β-cat, low E-cad) or yellow/orange (low β-cat, high E-cad).

Dissecting the single-crypt colonoid displayed in Fig. 3A–B to study the expression of different image sections showed a higher degree of heterogeneity at the “bottom” half of the colonoid. Orthogonal views in Fig. 3B showed a variety of expression, in particular, presence of bright “rings” of green (high membrane β-cat, low E-cad) stained cells bordered by clusters of “filled” green stained (high cytosolic β-cat, low E-cad) cells and “rings” of orange (high membrane E-cad) stained cells. Figure 3C shows a 2D montage of confocal sections for a large-body colonoid on day 7 constructed from adjacent fields of view, highlighting the size of these colonoids (>300 μm). Zooming in on one of the budding crypts (Fig. 3C boxed) and reconstructing the 3D structure (Fig. 3D), the heterogeneous expression of β-cat and E-cad (patches of cells in orange or green) can be observed on the surface of these colonoids. Figure 3E–F shows a montage of 2D images through the depth of the budding crypt in Fig. 3D and the orthogonal views shows a well-formed crypt had developed. Importantly for large-body colonoids, at sites of crypt bud formation, there were clusters of cells containing high levels of E-cad (cell clusters appearing bright orange/yellow, orange arrows in Supplementary Fig. S3F) surrounded by patches of β-cat high/E-cad low (bright green staining, green arrows in Supplementary Fig. S3F) cells, validating our previously published findings for the isolated colon crypts^26^.

### Regional β-cat distribution of *in vitro* colonoid crypts

To understand the Wnt activation activity of colon crypt cells with different β-cat and E-cad expressions, the nuclear β-cat levels of selected cells with different β-cat and E-cad membrane intensity in the colonoid crypts were analysed (Fig. 3G). These cells were pooled (n = 49 cells from four *in vitro* crypt buds, p > 0.05 between crypts using a one-way anova multiple comparison test) and categorized into 3 groups based on their β-cat/E-cad membrane intensity ratio (β/E), namely high (H, β/E > 75% percentile), low (L, β/E < 25% percentile) and medium (M, 75% percentile ≤β/E ≤ 25% percentile). The nuclear β-cat concentration of each of the selected cells was extracted computationally from the 3D image stacks using a 3D nuclear isolation algorithm (Fig. 3H) and tabulated against the respective membrane β/E ratio (Fig. 3I). Correlation analysis indicated a correlation between nuclear β-cat levels with the corresponding membrane β/E ratios (Fig. 3I, Pearson r = 0.48, p < 0.001). Tabulating against the respective membrane β/E category suggested that cells with a low β/E ratio (i.e. “L”, low membrane β-cat relative to E-cad) have significantly lower nuclear β-cat concentrations (Fig. 3J, p < 0.01, H vs L).

To further compare *in vitro* crypts with isolated crypts, the regional β-cat and E-cad levels along the length of *in vitro* crypts were measured and compared with that of isolated crypts^26^. The *in vitro* crypts (n = 10) were divided into 5 regions (Fig. 3K) and analysed quantitatively. Figure 3L shows the results for total β-cat and E-cad (normalized to mean intensity) for the 5 regions along the crypt. There is clearly significantly higher β-cat levels at the bottom of the crypt buds (p < 0.01 between middle and bottom). E-cad levels appear to be higher in region B1, just above the bottom of the crypt bud (p < 0.05). Along the length of the crypt bud, β-cat change is significant (p < 0.001) while there were no significant changes in E-cad noted. β-cat levels in all three compartments (nuclear, cytosol and membrane) are significantly higher at the bottom of the crypt buds (p < 0.05 between M and B2, Fig. 3M). This distribution of β-cat (total and compartments) along the length of the crypt is similar to that reported in isolated crypts^26^. The distribution of E-cad along the length of the crypt bud differs from that reported for isolated crypts (which decreases gradually towards the bottom), with a higher level found at the top (Fig. 3L, label T1) and just above the bottom region (Fig. 3L, label B1). This difference might be due to the different environment these cells are in contact with during development (Matrigel *in vitro* and myofibroblasts *in vivo*).

### Presence of differentiated cells indicates normal development of colonoid crypts *in vitro*

**Figure 4:**
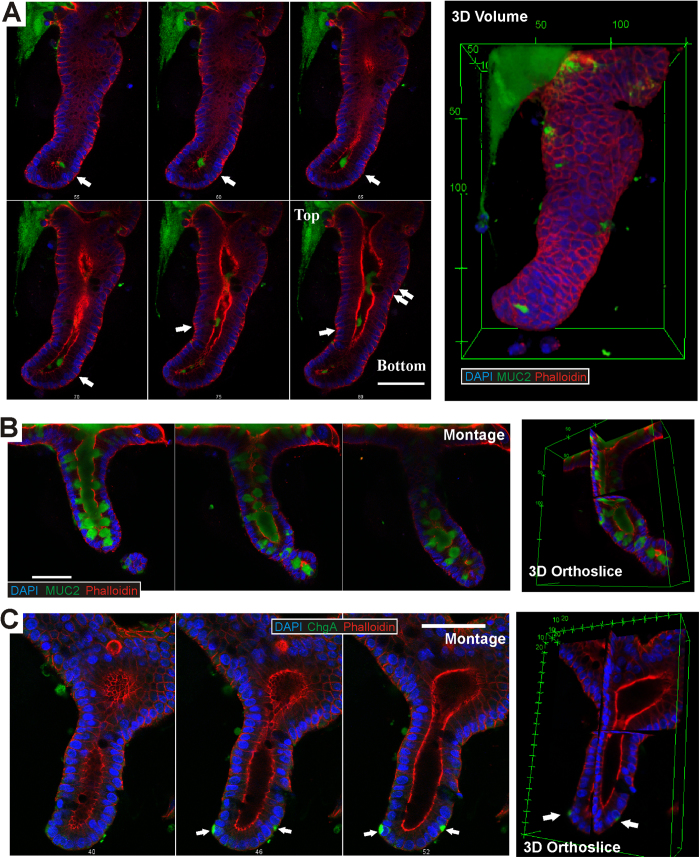
Mucin 2 and Chromogranin A expression in colonoids. Day 10 single-crypt and large-body colonoids were stained for DAPI (blue), Muc2 or ChgA (green) and F-actin (red). (**A**) The 2D montage showing the presence of various globlet cells along the length of the single-crypt colonoid with mucus secreting into the lumen (white arrows). 3D reconstruction of the colonoid (right panel) shows its similarity in shape to isolated crypts, with the top opening and crypt bottom clearly distinguishable. (**B**) 3D orthoslice view and 2D montage through the depth of a crypt budding off a large-body colonoid at day 10 stained for DAPI, Muc2 and F-actin are shown. The lumen and selected cells along the length of the crypt bud are filled with mucus. (**C**) 3D orthoslice views and 2D montage of a crypt budding off a large-body colonoid stained for DAPI, ChgA and F-actin, showing the presence of two ChgA positive cells in the bottom region of the colonoid crypt (arrows). Scale bars: 50 μm.

As verification that the colonoids were functionally normal and that the cells were growing and differentiating appropriately, the single-crypt colonoids were immuno-stained for Mucin 2 (Muc2)^27^. Muc2 is an indicator of the presence of globlet cells, a differentiated cell type of the colon crypt (Fig. 4A–B). Figure 4A shows a montage of 2D confocal images through the depth of a day 10 single-crypt colonoid where mucin can be seen secreting out of the globlet cells at various regions of the colonoid (white arrows in Fig. 4A). The colonoid had developed a near complete crypt shape with a top and bottom (Fig. 4A) and a distinct lumen lined by F-actin where the mucin was secreted into (this directionality is indicative of normal development). Figure 4B shows the montage of 2D confocal sections through a crypt budding from a day 10 large-body colonoid with the mucin (in green) present in cells along the length of the colonoid-crypt as well as in the lumen (see orthoslice views, right panel). Figure 4C shows the montage of 2D confocal images of a crypt budding from a day 10 large-body colonoid with two cells at the bottom of the crypt bud expressing Chromogranin A (ChgA, marker for enzyme secreting entero-endocrine cells^28^, white arrows in Fig. 4C). The expression of these two differentiation markers (Muc2 and ChgA) are similar to that observed in isolated colon crypts (Supplementary Fig. S2) and as reported by Sei *et al*.^29^ for duodenum crypts grown *in vitro*.

### Colon adenoma culture presents differences in development and molecular distribution of β-cat

**Figure 5:**
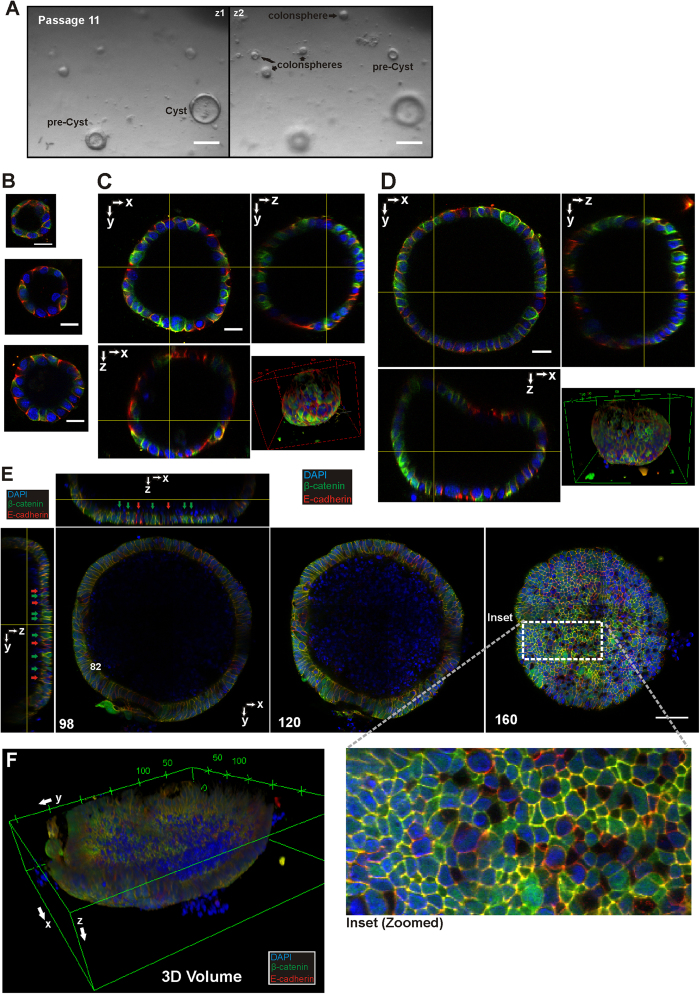
3D confocal imaging of colon adenoma cultures. (**A**) Bright field sectional images at different focal depths (z1 and z2) of colon adenoma cultures showing the different stages of development, namely from small colonospheres, increasing in size to pre-cyst (as the middle starts to collapse) and to a cystic structure. (**B**–**F**) Colonospheres and adenoma cysts are shown as RGB 2D images (blue:DAPI, green:β-cat, red:E-cad). (**B**) 2D overlay images of three small colonospheres increasing in size, showing the low frequency of distinct β-cat-high cells. (**C**–**D**) As the colonospheres increase in size, the number of β-cat-high cells increases in frequency. Orthoslice views and 3D reconstruction of two colonospheres (RBG overlays), showing the spherical shape of these structures and the gradual collapse of the top ceiling cell layer when dimensions increases (seen in D). (**E**) Orthoslice view and sectional 2D views (section 98, 120 and 160) of a large adenoma cyst showing how after collapsing, the structure flattens to form a “bowl” shape. Heterogeneous expressions of β-cat-high cells (green arrows) and E-cad-high cells (red arrows) are clearly identifiable along the surface of the cyst. (inset) A magnified region of the boxed (dotted) region of slice 160 shows clusters of β-cat-high cells and E-cad-high cells. (**F**) 3D reconstruction of the cyst showing the “bowl-like” shape of the structure. Scale bars: (**A**–**D**) 50 μm, (**E**) 100 μm.

**Figure 6:**
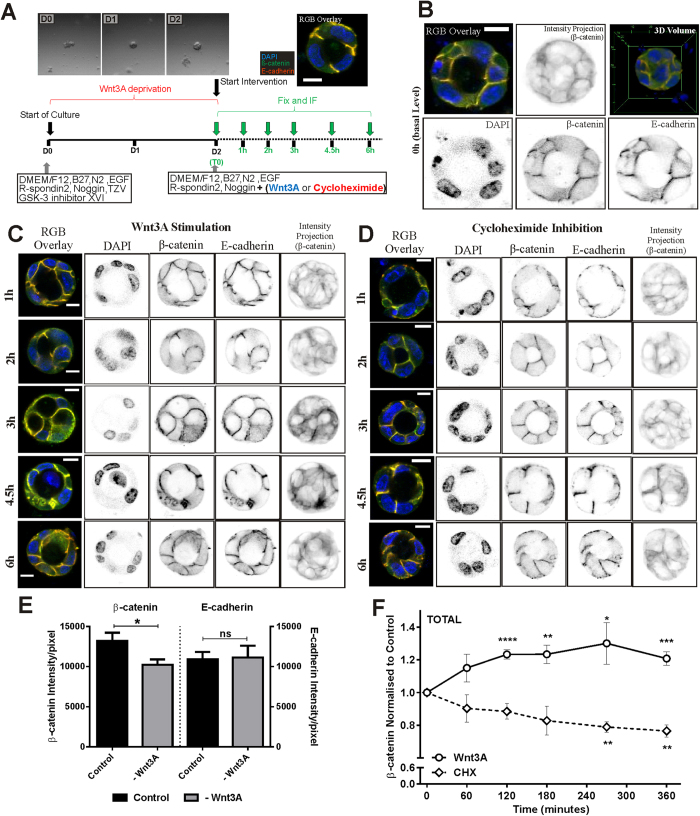
Quantitative analyses of total β-cat and E-cad dynamics in colonospheres. (**A**) Schematic of colonosphere perturbation experiment. A combination of GSK3β inhibitor XVI and TZV were added at day 0 with the standard medium without Wnt3A. Recombinant Wnt3A or CHX was added from day 2 for pre-defined durations, fixed and stained for β-cat and E-cad before imaging under the confocal microscope. (**B**) Base levels of β-cat and E-cad in a typical Day 2 colonosphere. Selected colonosphere are shown as RGB overlays of 2D images, intensity projection of 3D β-cat signal, 3D reconstruction and 2D sections of DAPI, β-cat and E-cad. Colonospheres of different duration and under (**C**) Wnt3A stimulation or (**D**) CHX inhibition are shown as RGB overlays, 2D sections of DAPI, β-cat and E-cad as well as the intensity projection of 3D β-cat. From the intensity projections, β-cat intensity increased under 3 or 4.5 hours of Wnt3A stimulation while changes were less clear for CHX inhibition. (**E**) Average total β-cat and E-cad intensity/pixel of positive control (+Wnt3A) and basal levels (-Wnt3A) showing a significant reduction in β-cat at the basal level during two days of Wnt3A deprivation while having no effect on the E-cad levels (*p < 0.05, ±SEM). (F) Relative total β-cat intensity quantitation of colonospheres under Wnt3A stimulation or CHX inhibition for 1,2,3,4.5 or 6 hours were tabulated. β-cat intensity increased significantly under Wnt3A stimulation (n = 5) while under CHX inhibition (n = 3) a gradual decrease in β-cat was observed, significantly after 4.5 hours. (relative to T_0_: * p < 0.05, ** p < 0.01, *** p < 0.001, **** p < 0.0001, ± SEM). Statistical tests: single-factor anova. Total colonospheres count:296. Scale bars: (**B**–**D**) 10 μm.

This colon culture setup was also applied in culturing crypts isolated from adenoma regions of APC^min/+^ mouse colon. In culture, colon adenoma do not form crypts and appear as either colonospheres or cyst-like spheres/discs in both the primary culture and subsequent passages (Fig. 5A). The adenoma colonospheres and cysts were immuno-stained for β-cat and E-cad and 3D image stacks were acquired. Adenoma colonosphere and cyst confocal images are shown as overlayed images (blue: DAPI, green: β-cat and red: E-cad) in Fig. 5B–F. Figure 5B shows early adenoma colonospheres as spheres and the distribution of β-cat and E-cad in these early cultures. Most cells in the colonospheres expressed E-cad, however only a few cells were positive for β-cat, unlike the uniform distribution observed in early colonospheres derived from C57BL/6 mouse (Fig. 6). The frequency of these β-cat expressing cells appears to increase with colonosphere size (Fig. 5C–D). The cross-sectional views of these colonospheres shows that they maintained their spherical structure until they reach a certain size (for the colonosphere in Fig. 5D, diameter of 167 μm) after which the top section appears to flatten towards the base (Fig. 5D), eventually forming a cyst-like structure (Fig. 5E). Figure 5E is a RGB overlay montage through the depth of an adenoma cyst showing the heterogeneous expression of β-cat and E-cad around the cyst. Patches of β-cat filled cells (green) as well as cells with high levels of E-cad (orange) can be clearly seen (inset of slice 160) at the bottom of the cyst. The cross sectional views of the cyst (Fig. 5E) show that it is has a flat bottom, one to two cells thick with heterogeneous clusters of β-cat and E-cad expressing cells (green and red arrows). 3D reconstruction of the adenoma cyst (Fig. 5F) further demonstrates the flattened shape of the cyst (Supplementary Video S3). Analyses of the β-catenin compartment ratios of adenoma colonoids showed a lower cytosolic β-catenin ratio as compared to that of normal C57 derived colonospheres (at T0) with the nuclear and membrane compartment ratios at comparable levels with that of the normal colonospheres (Supplementary Figure S4A). These differences might be attributed to the lower number of β-catenin filled cells in the adenoma colonospheres (see Fig. 5B and Supplementary Figure S4B). For the β-catenin filled cells, 3D compartment analyses further suggest mainly membrane or nuclear localisation with the low levels of cytosolic β-catenin appearing to be homogeneously distributed (Supplementary Figure S4C).

### Quantitative analysis of the effects of Wnt3A and cycloheximide on β-cat and E-cad levels in colonospheres

In order to quantitatively analyse the dynamics of Wnt signalling under Wnt3A stimulation in the colon crypt cells, we depleted the cultures of Wnt3A ligand for 2 days (Fig. 6A). To maintain the viability of small colonospheres for the duration of Wnt3A deprivation, GSK3 Inhibitor XVI and E-cad stabilizer (Thiazovivin, TZV) were added to the cultures (Fig. 6A). TZV is a potent ROCK inhibitor and stabilizes newly synthesized cell surface E-cad and cell-cell adhesion, decreasing anoikis and promoting stem cell survival^30^,^31^. The GSK3 inhibitor promotes survival of small intestinal stem cells during the first 2 days of culture in the absence of Wnt3A^31^. Using this setup, colonospheres were present from day 1 (Fig. 6A, inset). The day 2 colonospheres (Wnt deprived) were stimulated with Wnt3A or inhibited with cycloheximide (CHX) for 0, 1, 2, 3, 4.5 or 6 hours respectively (Fig. 6A). CHX (a protein synthesis inhibitor) was used to study the degradation dynamics of β-cat and E-cad. The distribution and levels of β-cat and E-cad in these colonospheres was quantitated by 3D confocal microscopy. There was a significant reduction in β-cat to the basal level after the two days of Wnt3A deprivation (comparing T_0_ and positive control, p < 0.05) while two days of Wnt3A deprivation did not affect the E-cad levels significantly (Fig. 6E).

Figure 6B–D shows confocal 2D images of colonospheres treated with Wnt3A or CHX for the respective time durations, stained with DAPI (marking the nucleus), β-cat and E-cad. The 3D image stack encompasses the entire colonosphere and the intensity of β-cat in each colonosphere is shown as intensity projection maps of the 3D image stack as a visual indicator of 3D β-cat levels. The projected signal intensity map of β-cat was obtained by summing and then averaging the intensities through the depth of the image to give a 2D representation of the 3D signal intensity of the colonosphere. Over 200 (total from n > = 3 experiments) colonospheres were imaged and the relative total β-cat and E-cad intensities at each time point were quantitatively analysed (Fig. 6E–F). Wnt3A stimulation stabilizes β-cat levels in the colonospheres with an increase of 20% above the basal level after 3 hours of Wnt3A stimulation (Fig. 6C,F). The Wnt3A stimulated β-cat after 3 hours was similar to the β-cat levels of the positive control (stimulated by Wnt3A for two days, Fig. 6E). Inhibiting protein synthesis with CHX caused the β-cat levels to decrease by 20% after 4.5 hours of inhibition (Fig. 6D and E, p < 0.01), a significant level of degradation in the colonospheres.

### Quantitative analysis of compartment β-cat and E-cad in colonospheres

**Figure 7:**
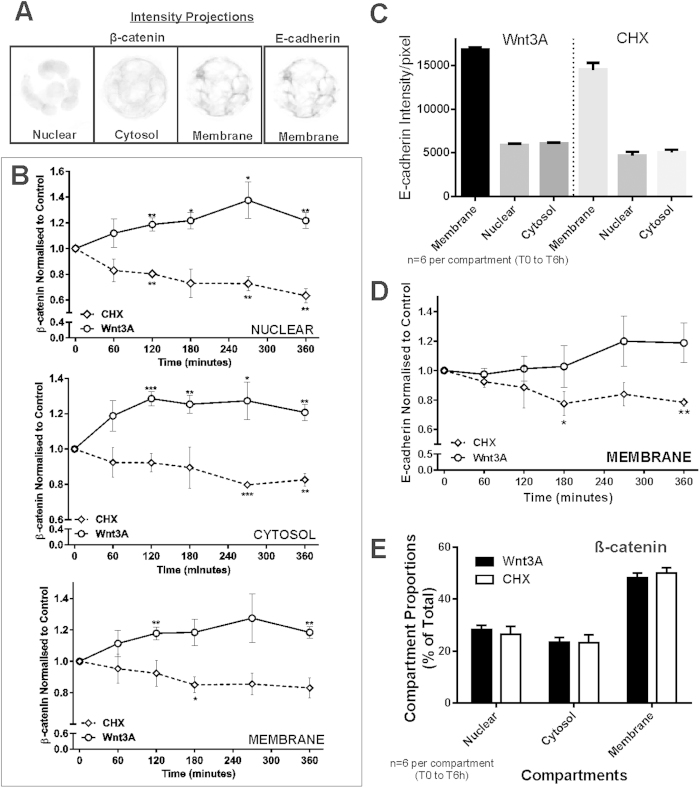
Quantitative analyses of compartment β-cat and E-cad dynamics in colonospheres. (**A**) Intensity projections of 3D β-cat (nuclear, cytosol or membrane compartments) and E-cad (membrane only) of day 2 colonospheres. (**B**) Relative subcellular compartment β-cat intensity of colonospheres under Wnt3A stimulation or CHX inhibition (1, 2, 3, 4.5 or 6 hours). Nuclear β-cat increased significantly after 2 hours of Wnt3A stimulation and decreased significantly after 2 hours of CHX inhibition. Relative cytosolic β-cat increased significantly after 2 hours of Wnt3A stimulation while under CHX inhibition, a slow decrease was observed which became significant after 4.5 hours of inhibition. Relative membrane β-cat increased slightly under Wnt3A stimulation (significant after 2 hours of stimulation) with a very slow decrease observed under CHX inhibition. (**C**) Average compartment E-cad intensity (all T_0_ to 6 hrs) for either Wnt3A or CHX treatment. The nuclear E-cad intensity detected was treated as background control (the antibodies used recognize only E-cad extracellular domain). In both treatments, the average E-cad intensity/pixel for both nuclear and cytosol compartments are similar, rendering cytosolic E-cad intensity detected as background. E-cad analyses therefore only consisted of the membrane compartment (minus the corresponding nuclear intensity as background correction). (**D**) The relative E-cad levels (background corrected) under Wnt3A stimulation or CHX inhibition. E-cad levels increased variably by 20% (p > 0.05 for each time-point) after 6 hours of Wnt3A stimulation. CHX decreased the E-cad levels by 20% after 3 hours (p < 0.05). (**E**) Compartment proportions (% nuclear, cytosol or membrane) of β-cat intensities were tabulated for all colonospheres under the two treatments (T_0_-T6 h). No change in compartment proportions was observed under either Wnt3A or CHX perturbations. Wnt3A n = 5, CHX n = 3, statistical test: single-factor anova analysis relative to T_0_: *p < 0.05, **p < 0.01, ***p < 0.001. Total colonospheres count:296. Error bars: ± SEM

We measured the β-cat and E-cad immunofluorescence levels in the different subcellular compartments. Figure 7A shows the typical intensity projection maps of compartment (i.e. nuclear, cytosol and membrane) levels of β-cat and E-cad (membrane compartment) in colonospheres. Figure 7B tabulates the relative β-cat compartment levels of over 200 colonospheres (with respect to basal intensities). This quantitation show that nuclear β-cat levels increased significantly with Wnt3A stimulation (Fig. 7B, after 2 hours of stimulation) and decreased upon CHX inhibition (Fig. 7B, after 2 hours inhibition). Cytosolic β-cat levels increased with Wnt3A stimulation (after 2 hours) and decreased gradually in the presence of CHX inhibition (significantly after 4.5 hours). Membrane β-cat levels increased with Wnt3A stimulation to about 20% of basal levels after 2 hours of stimulation. In the presences of CHX inhibition, membrane β-cat levels decreased slowly to about 15–17% lower than basal level after 3 hours of inhibition.

E-cad should not be detected in the nuclear (the antibodies used in this experiment only recognizes E-cad extracellular domain), so the nuclear intensity is treated as background signal. Figure 7C shows the intensity of E-cad signal quantitated in the different compartments. In both Wnt3A treated and CHX treated colonospheres, the average E-cad intensity (across the 6 time points) in both the nuclear and cytosol compartments are similar. Therefore, there was no cytosolic E-cad detected in these colonospheres. Subsequent E-cad analyses used the corresponding nuclear signal for background correction. Figure 7D shows the results for relative E-cad levels (membrane intensity with background correction, relative to basal intensities). The quantitation showed that E-cad levels increased slowly after 4 hours and variably (p-value > 0.05 for each time-point) under Wnt3A stimulation to 20% of basal level after 6 hours. Presence of CHX decreased the E-cad levels to 20% of its basal level by 6 hours.

Analysis on β-cat distribution among the compartments throughout the 6 hour experiment (in terms of the proportions of β-cat amongst the compartments) was conducted. Surprisingly the average compartment proportions of β-cat remained stable under either Wnt3A stimulation or CHX inhibition (Fig. 7E) for the duration of the perturbation.

## Discussion

### A colon crypt culture system for studying the role of molecular signalling

This study introduces an integrated 3D imaging and colon culture system suitable for both cell biological and molecular analyses. Consequently we have acquired quantitative data of protein dynamics during crypt development. Previously, colon cultures only formed new crypts (i.e. colonoids) using a support layer of colon subepithelial myofibroblasts^23^. By using a new culture setup, i.e. plating the epithelial cells on the Matrigel without the support cells, single colon crypts formed rapidly (in 3 to 4 days) and new crypts form from colonospheres within 10 days. This culture setup eliminates the possibility that specific regulators are acting on the support cells and indirectly affecting the development of the colon epithelial cells. It should be noted that normal colon crypt epithelial cells cannot grow as a monolayer in cultures, they die. Matrigel is needed in 3D cultures to provide the support, however the use of Matrigel causes complications for immuno-staining and other intervention applications (e.g. the transfection success rate is poor when cells are embedded in Matrigel^32^). The new setup resolved this issue as the crypt cells grow on top of the Matrigel. This setup not only allows direct stimulation of the colon cells via bioactive stimuli in the medium, it facilitates removal of culture debris during medium changes. This culture setup will also facilitate the study of interventions such as the knockdown of particular target genes (utilizing technologies like siRNA or CRISPR/cas9^33^) and their effect on crypt development and molecular signalling.

### Studying of colon crypt development mechanisms, niche and markers *in vitro*

By following the colon culture daily, we detected two mechanisms of crypt formation. The semi-digested crypt fragments rounded up into small colonospheres by day two. Some of these colonospheres proceeded to either grow in size or flatten out as a cyst-like colonosphere/disk until they formed new crypts (large-body colonoids). This crypt formation process also occurs in previous culture methods^19^,^23^ and typically started after day 6 (Fig. 1G). On the other hand, some of the day two colonospheres formed single crypts with a lumen opening on one end and a distinctive crypt bottom at the other end (single-crypt colonoids). This crypt formation process was observed earlier (day 4) compared to the large-body colonoids, which appear after day 6 (Fig. 1F). These colon crypt growing *in vitro* are good representations of the colon crypts *in vivo* as they have similar morphology, differentiated cell expressions and β-cat regional distribution (Fig. 3) as that measured in isolated colon crypt previously reported by our laboratory^26^.

It is still unclear whether these two different mechanisms came about due to environmental influences or because of the composition of the cells which initiated the colonosphere. Factors influencing whether these colon crypt forming events eventually succeed in creating new crypts are still unclear. One factor we tested is the effect of noggin on colon crypt formation. Noggin (an antagonist of BMP signaling^34^) is known to inhibit the action of BMP signalling, which in turn inhibits intestinal stem cell self-renewal by suppressing Wnt signaling^35^. By withdrawing noggin from the colon culture on day 2, the proportions of single-crypt colonoids formation increases, implying a role of BMP signalling for initiating the formation of the crypt shaped structure *in vitro*.

The key difference between the new colon culture and the traditional 3D Matrigel culture^19^,^23^ was that the colon crypt fragments were not embedded in Matrigel but were suspended in the culture medium and allowed to settle on top of the Matrigel (Fig. 1A). The Matrigel was plated in such a way that a Matrigel gradient was created in the well (i.e. thicker around the edge and falling away toward the centre of the well). This change in the physical environment was sufficient to facilitate crypt formation events, particularly the observation of single-crypt colonoids, highlighting the importance to regulate and control the environmental niche of the culture.

This setup opens up an opportunity for interrogations of stem cell markers and roles of different cell types in colon crypt development. Current work on the colon was limited due to the inability to efficiently grow colon crypts *in vitro*. Most work on the intestinal cultures has been conducted in small intestine cultures based on culture techniques established by Sato and Clevers *et al*.^19^,^21^. They established the small intestine crypt cultures with the growth of self-organizing mini-guts *in vitro* (as reviewed in ref.^36^). They described crypt formation events as “symmetry-breaking” events due to the sporadic appearance of a paneth cell in a symmetrical cyst like structure. A bud forms around the cell and developed a crypt-like structure with stem and paneth cells, potentially driven by EphB-EphrinB interactions^36^. However, this hypothesis cannot be translated to the colon as it does not have paneth cells, raising the question: what drives the “symmetry-breaking” event in the colon? Clearly further work will be necessary to clarify this question and our colon culture system will be able to facilitate such interrogations.

### Integrating colon culture and animal models enables systems biology of CRC

Integrating this culture setup with animal models enables colon crypt development and protein dynamics of mice from different genetic backgrounds to be studied. Specifically, the differences in cellular signalling and morphological development of colon crypts due to specific mutations (e.g. APC truncation) can be systematically elucidated. Morphological analyses of crypts isolated from either C57BL/6 mice or Tg(A33-CreERT2);APC^fl/fl^ mice (*in vivo* Tamoxifen induction to induce APC truncation) suggested differences in morphology during colon crypt development (Supplementary Fig. S3). Culturing colon adenoma crypts isolated from the adenoma regions of the colon from APC^min/+^ mice has also shown clear differences in development (Fig. 5A) and spatial distribution of β-cat and E-cad (Fig. 5F). Colon adenoma cultures do not form crypts *in vitro* and express localized clusters of cells with high levels of β-cat or E-cad. These initial observations are different to the colon cultures from C57BL/6 mice (Fig. 6B–D)and further quantitative measurements and comparisons between these normal and adenoma cultures will provide new information on the effects of molecular aberrations on colon development *in vitro* as well as changes to the molecular dynamics of key proteins. Using the APC^min/+^ mouse model to establish colon adenoma cultures as a basis for drug discovery studies is clinically relevant as it is a pure adenoma model with well-defined genetic etiology closely mimicking the truncation of the APC gene as observed in most sporadic human colon adenomas.

3D quantitative imaging and in-well staining allows spatial and temporal location of key signalling molecules to be visualized, measured and analysed and the use of C57BL/6 colon crypt culture as the baseline system enables different animal models to be compared and quantitated. This setup will be useful for conducting targeted testing of therapeutic interventions/strategies *in vitro* to significantly reduce the number of animal xenograph experiments required during clinical trials.

This study introduces a systems biology approach to elucidating CRC for computational prediction, target validation and *in vitro* drug testing in a biological system of relevance to CRC. The system can be quickly applied and translated into human colon tissues, which will be the critical next step to understanding the mechanism of the disease and to the development of new therapeutics for CRC.

## Methods

### Animals

All experiments were performed in accordance with relevant guidelines and regulations approved by the Walter and Eliza Hall Institute Animal Ethics Committee (approval number 002/11). C57BL/6 mice were maintained in the animal facility at WEHI Bioservices and bred under specific pathogen-free conditions. APC^min/+^ and Tg(A33-CreERT2);Apc^fl/fl^ mice colon kindly provided by Dr Anuratha Sakthianandeswaren and Dr Michael Buchert (The Walter and Eliza Hall Institute of Medical Research, Victoria, Australia).

### Reagents used for colonoid culture

The combinations of growth medium and reagents used in this study were as follows.

#### Basic Medium

DMEM-F12 (Life Technologies, #10565018) supplemented with penicillin-streptomycin (Life Technologies, #15140-122), 1x GlutaMAX (Life Technologies, #35050-061), 1x N2 (Life Technologies, #17502), 1x B27 (Life Technologies, #17504) and HEPES (10 mM).

#### Standard Growth Medium

Basic Medium, R-spondin-Fc Conditioned Medium* (2%), recombinant Wnt-3A (100 ng/mL, PeproTech, # 315-20), recombinant human Noggin (100 ng/mL, PeproTech, #120-10), recombinant mouse EGF (50 ng/mL, PeproTech, #315-09) and Rho-kinase inhibitor Y27632 (10 μM, Sigma-Aldrich, #Y0503).

#### Wnt3A Starvation Growth Medium

Basic Medium, R-spondin-Fc Conditioned Medium* (2%), GSK-3 Inhibitor XVI (3 μm, EMD Millipore, #361559), iPSC Induction Enhancer Thiazovivin (3 μM, TZV, EMD Millipore, #420220), recombinant human Noggin (100 ng/mL, PeproTech, #120-10), recombinant mouse EGF (50 ng/mL, PeproTech, #315-09) and Rho-kinase inhibitor Y27632 (10 μM, Sigma-Aldrich, #Y0503).

#### Perturbation Medium

Basic Medium, R-spondin-Fc Conditioned Medium* (2%), recombinant human Noggin (100ng/mL, PeproTech, #120-10), recombinant mouse EGF (50ng/mL, PeproTech, #315-09). Either recombinant Wnt-3A (100 ng/mL, PeproTech, # 315-20) or cycloheximide (CHX, 100μg/ml, Sigma-Aldrich, #C4859) was added depending on the perturbation required.

#### Digestion mix

0.1 mg/mL of Dispase (Life technologies #17105-041) in DMEM-F12.

*R-spondin-Fc Conditioned Medium was harvested from 293F cells (after 7 days) transiently transfected with a human R-spondin2 construct that has Fc-fusion protein fused to the C-terminus and cloned into pApex vector. Note that Y27632 are only added during the first four days of all colonoid cultures.

### Intestinal colon crypts isolation, preparation and culture setup

**Isolation of colonic crypts** (Modified from ref.^23^): The distal half of a colon (including the rectum, excluding the cecum) was removed from the mouse and cut opened in a longitudinal direction using a pair of dissection scissors, and kept on ice. The colon was washed with 10 mL of ice-cold PBS and then treated with 0.04% (wt/vol) sodium hypochlorite in PBS at room temperature for 5 min. The colon was washed with PBS and incubated with 5 ml of chelation buffer (PBS with EDTA 3mM and DTT 0.05 mM) for 60 min at room temperature. The colon was washed with PBS, suspended in 2 ml of PBS, and shaken vigorously for 10 seconds to release crypts. The supernatant was collected. This crypt isolation step was repeated 5 times. The supernatant, which was rich in isolated crypts, was centrifuged at 100 g for 1 minute to remove single cells. Essentially the pellet contained pure crypts.

#### Partial crypt fragments preparation

The crypt pellet was re-suspended in 3mL of the *digestion mix* and incubated at 37 ˚C for 10 minutes. The suspension was then shaken vigorously and pipetted with a 1000 μL pipette tip about 10 times before incubating at 37 ˚C for a further 10 minutes. The suspension was then pipetted 10 times and centrifuged at 100 g for 1 minute. The supernatant was discarded and the pellet was re-suspended in 3 mL of DMEM-F12. The suspension was centrifuged at 100 g for 1 minute. The supernatant was discarded and the pellet (containing partial crypt fragments) was re-suspended in 100 μL of DMEM-F12. The number of partial crypt fragments was counted by a haemocytometer and the concentration adjusted to 200 pieces /100 μL with the appropriate medium (either *Standard Growth Medium* or *Wnt3A Starvation Growth Medium*). The medium used depended on the nature of the experiments.

#### Colon Crypt Culture setup

The required wells of a 96 well plate (either conventional 96 well plate (BD Falcon, # 353072) or Corning^®^ 96 well special optics plates (Corning, #3720)) was coated with 15 μL of BD Matrigel™ matrix (BD Bioscience, #356237), making a ring along the circumferences of the well as shown in Fig. 1A. The plate was incubated at 37 ˚C for 30 minutes to allow Matrigel to polymerize. 100 μL of the partial crypt fragment suspension (containing 200 pieces) was added to each well (dropping the suspension vertically down into the middle of each well) and incubated at 37 ˚C. The medium was replaced every two days. For daily monitoring of colon culture morphological changes during crypt development, the partial crypt fragments were suspended in the *Standard Growth Medium*. For specific withdrawal experiments including investigation of the effects of GSK-3 Inhibitor XVI and TZV as well as Wnt3A withdrawal experiments, the partial crypt fragments were suspended in *Standard Growth Medium* with/without the reagent(s) of interest. For experiments to measure β-cat dynamics in colonospheres under Wnt3A and CHX perturbations, the partial crypt fragments were suspended in *Wnt3A Starvation Growth Medium* (see section below):

### Intestinal colon adenoma crypts isolation, preparation and culture

Adenoma tissues was resected from the colon of an APC^min/+^ mouse and washed with 5mL of ice-cold PBS. After treating with 0.04% (wt/vol) sodium hypochlorite in PBS at room temperature for 10 minutes, the adenoma was washed with PBS and incubated in a tube containing 5 mL of chelation buffer (PBS with EDTA 3 mM and DTT 0.05 mM) for 30 minutes at room temperature. During the incubation, the tube was occasionally shaken vigorously to remove surrounding crypts from the adenoma. The adenoma was cut into small pieces using a scalpel blade and suspended in 1 mL of the **digestion mix** and incubated at 37 ˚C for 20–30 minutes.

The suspension was shaken vigorously and pipetted with a 1000 μL pipette tip 10 times and incubated at 37 ˚C for a further 5 minutes. The suspension was pipetted with a needle (G26) 20 times and centrifuged at 800G for 2 minutes. The supernatant was then discarded and the pellet re-suspended in 1 mL of fresh DMEM-F12. The suspension was centrifuged at 800G for 2 minutes, after which the supernatant was discarded and the pellet (which contains adenoma fragments/cells) re-suspended in 100 ~ 200 μL (the volume used is dependent on the size of adenoma) of **Standard Growth Medium**. 100 μL of the suspension was added to each well pre-coated with Matrigel (see “**Colon Crypt Culture setup**”) and incubated at 37 ˚C. The medium was replaced every two days.

### Crypt culture Microscopy and Image Processing

Bright field microscopy was conducted using a Nikon Eclipse Ti-U microscope with a 4x objective lens. For each well of the culture, twelve *fields of view* (FOV) were imaged in a predefined order (in this study: grid wise, snake by rows, from left then down. See Supplementary Fig. S1A). The depth of the crypts and Matrigel™ layer were acquired with multiple images taken for each FOV at different depth/focus (see Supplementary Fig. S1B). The images were captured on a Photometrics CoolSnap HQ CCD camera (Roper Scientific) using the ImageJ/Fiji software with the micro-manager software (version 1.3). The output analogue signal was digitized and saved as tiff images with correlating images for each FOV stack and organized into folders. At experiment specific time points (e.g. 1, 2, 3, 4 and 5 days), the whole well was imaged (encompassing the twelve FOV) to acquire time-lapse images of the crypts as they formed spheroids and colonoids. Each dataset (specific to each time-point and perturbation) consists of 12 FOV folders, each consisting of 2D images at different depths/focus. The datasets were loaded using ImageJ/Fiji software and processed. The images in each FOV folder were organized into an image stack and compressed using the “Extended depth of field” plugin^37^ into a 2D image representation of the images with the following customized settings (see Supplementary Fig. S1). Each compressed 2D image (2D-EDOF) was named after the FOV number on the grid (see Supplementary Fig. S1). The twelve 2D-EDOF images were then processed using the “Grid/Collection Stitching” plugin with the customized settings (Supplementary Fig. S1) to create one complete image of the culture well. A selection of images was generated using a 15% to 35% tiling overlap between the FOVs then the best reconstructed image was visually selected. This process from the raw acquired images to the final complete culture well images has been automated with a Fiji/ImageJ script (see Supplementary Fig. S1). These images can then be used for analyses: colonospheres/colonoids monitoring, culture efficiency, quantification and morphology analyses.

### Perturbation of colonospheres in culture with Wnt3A and CHX

For experiments measuring β-cat dynamics in colonospheres, the colon crypts were plated in Corning^®^ 96 well special optics plates (Corning, #3720)). As mentioned previously, at the start of the colon culture, the *Wnt3A Starvation Growth Medium* was used to culture the colon (This mixture contains TZV and GSK3 inhibitor XVI to stabilize the culture while starving the culture of Wnt3A stimulation). The positive control wells were the only ones with the *Standard Growth Medium*. The culture was allowed to grow for two days before the start of the perturbation time course. At the commencement of the time course, the selected culture wells for perturbation were incubated with fresh *Perturbation Medium* (which either contains Wnt3A or CHX) for 1, 2, 3, 4.5 or 6 hours at 37 ˚C. The control wells had their medium changed with fresh *Wnt3A Starvation Growth Medium*. After the incubation, the cultures were fixed and stained in-well for β-cat and E-cad as described in the next section.

### Immunofluorescent staining of colonospheres, colonoid and adenoma culture

This protocol was adapted from Tan *et al*.^26^ methods for isolation of crypts and applied to the colonospheres/colonoids/adenoma cysts, staining was in-well. The medium was removed from the wells and the culture was washed twice with 150 μL of PBS per well. The PBS was removed and 150 μL of 0.1% Paraformaldehyde (PFA) in PBS was added to each well and incubated at 4 ˚C for 48 hours. The paraformaldehyde was removed from the wells and individual wells were washed with 150 μL of PBS twice. The PBS was removed and 150 μL of 0.2% Triton X-100 in PBS (vol/vol) was added into each well (for permeabilization) and incubated for 20 minutes at room temperature. 150 μL of blocking buffer [0.2% (wt/vol) bovine serum albumin (Sigma Aldrich #A7030) in PBS] was added to each well and incubated for 12 hours at 4 ˚C. 30 μl of the primary antibody solution [0.2% BSA (w/v) in MT-PBS, rabbit anti-β-cat (Sigma-Aldrich #C2206, 1:200), and rat anti-E-cad (Invitrogen #13–1900, 1:200)] was added to each well and incubated at 4 ˚C for 12 hours. The solution was removed and 150 μL of blocking buffer (0.1% (v/v) TritonX-100 in MT-PBS) was added into each well and incubated for 20 minutes at room temperature. The blocking buffer was removed and 30 μl of secondary antibody solution (0.2% BSA (w/v) in MT-PBS) added containing Alexa Fluor® 546 goat anti-rat IgG (H+L)(Molecular Probes Inc, Eugene, OR, Invitrogen cat#A-11081,1:200) and Alexa Fluor^®^ 488 goat anti-rabbit IgG (H+L) (Molecular Probes Inc, Eugene, OR, Invitrogen cat#A-11034, 1:200) to each well. 50 μL of 1nM DAPI (4, 6-diamidino-2-phenyl indole Nucleic Acid Stain, cat# D1306 Molecular Probes Inc, Eugene, OR, Invitrogen) in MT-PBS was added to each well and incubated for 15 minutes at room temperature. Remove the DAPI solution and add 50 μL PBS to each well. If the plate used is a Corning^®^ 96 well special optics plate (Corning, #3720), the colonoid culture is now ready for 3D confocal fluorescence microscopy. If the colonoid culture is to be stained in a conventional 96 well plate, the colonoid cultures are harvested from the wells and mounted onto optical cover slips and slides. To minimize the distance between the colonoid samples and microscope lens (as the working distance for high resolution lens is less than 200 μm) to prevent compression of the colonoids which could change their shape or sizes, a customized coverslip and slide setup was utilized. Two strips of double sided tapes was used to create a “channel” while holding the coverslip and slide together. One side of the “channel” is firstly sealed by nail polish before the stained colonoid solution is pipetted into this “channel” from the open end. The open end is then sealed with nail polish, creating a thin channel of colonoid sample. The mounted sample is now ready for 3D confocal imaging. The samples were stored in the dark at 4 ˚C before imaging.

For Muc2 and ChgA immunofluorescence, either 0.2% BSA (w/v) in MT-PBS of rabbit anti-Mucin 2 (H-300, Santa Cruz Biotechnology, #sc-15334, 1:50) or rabbit anti-Chromogranin A (ImmunoStar Inc, #20086, 1:50) primary antibodies were used with Alexa Fluor^®^ 488 goat anti-rabbit IgG (H+L) (Molecular Probes Inc, Eugene, OR, #A-11034, 1:200) as the secondary antibody and Alexa Fluor^®^ 546 Phalloidin (Molecular Probes Inc, Eugene, OR, #A-22283,5 units/mL) to stain the F-actin.

## 3D Confocal Fluorescence Imaging. 

Immunofluorescent staining of the colonospheres and colonoids was detected with an Olympus FV1000 Spectral Confocal attachment to an Olympus IX-81 microscope on a 60x water immersion lens (for spatial analysis of proteins). The crypts were imaged using standard filter sets and laser lines, acquiring single labelled images. DAPI, (β-cat or Muc2 or ChgA) and E-cad fluorescence was excited with the 405 nm, 488 nm and 546 nm laser lines respectively, and the emission wavelength were measured at wavelengths 405nm, 473 nm and 559 nm respectively. The images were captured using Olympus FluoroView software (Version 1.7c). 3D image stacks were acquired which encompassed the entire depth of the colonoids in the field of view. Cubic voxels were acquired for each image stack and the output analogue signal, representing the fluorescence intensities digitized to 16 bits resolution at 65536 levels of grey and saved as an Olympus Image Binary (OIB) image. The images were imported into the ImageJ/Fiji software^38^ for processing and visualization using Bioformats plugins.

For quantitative 3D imaging of colonospheres, a minimum of 3 colonospheres (typically >4) were imaged per perturbation, per time-point (including positive and negative controls) for each experiment with at least 3 independent experiments conducted. Small round colonospheres (diameters between 25 to 45 μm) were typically present at this stage of the culture and imaged. 3D image stacks of 296 colonospheres were acquired by stacking hundreds of sequential 2D images for each colonosphere. In this study, either Wnt3A or the inhibitor CHX was added to the colon culture and spatial 3D image stacks acquired 0, 1, 2, 3, 4.5 or 6 hours after adding the reagent.

These image stacks were then processed using ImageJ/Fiji image processing software and Matlab (R2011b) to obtain the resultant β-cat and E-cad quantitation for each colonosphere (see the next section).

### Image Processing and Analyses

Quantitative analyses including total and compartmental β-cat and E-cad concentrations (intensity per pixel) in colonospheres and along the length of colonoid crypts as well as β-cat intensity concentration of individual nucleus in each colonoid crypt (nuclear β-cat isolation) were conducted on 3D image stacks of colonoids and colonospheres acquired (as described in the previous section). These 3D image stacks consists of 2D images detecting immunofluorescent staining of DAPI, β-cat and E-cad. A customized ImageJ/Fiji macro was written to convert Olympus OIB image files to TIFF files (Tagged Image File Format), which is readable by most image processing software. A customized Matlab script was written which loads and process these 3D image stacks. The Matlab script was modified from the methods used in our previous work^25^ with improvements made to advance to a three compartment analysis and to isolate individual nuclear in 3D^26^. The customized Matlab script used for the analyses is available upon request.

#### Colonoid quantitation

3D image stacks of Day 2 colonoids were obtained for n > 3 experiments. Image processing and quantitative compartmental angalysis was performed on the resulting data files (using a customized Matlab script) to obtain the resultant β-cat and E-cad measurements for each colonosphere.

#### in vitro colonoid crypts quantitation

For *β-cat regional compartment quantitation*, *in vitro* colonoid crypts (n = 10) were extracted from 3D image stacks of colonoids acquired (in the previous section). Each colonoid crypt image stack was subdivided along the length into five regions (see Fig. 3J), extracting each region as an individual image stack. Image processing and compartmental analyses were conducted (using the Matlab script) on each regional stack and the resultant β-cat and E-cad measurements for each colonoid crypt were obtained and tabulated.

For measuring the *β-cat intensity concentration of individual nucleus* in each colonoid crypt (Nuclear β-cat intensity isolation), *in vitro* colonoid crypts (n = 4) were extracted from the 3D image stacks of acquired colonoids. Image processing and compartmental analyses was conducted (using the Matlab script) on each colonoid crypt 3D stack. Individual nucleus in each colonoid crypt was extracted and the position and β-cat concentration (intensity per pixel) tabulated and visualized (see Fig. 3H). From the colonoid crypts processed, n > 12 cells with distinct membrane localization of E-cad and/or β-cat were visually selected by going through the image stacks manually. A segmented line of interest was drawn along the membrane of these cells (see Fig. 3G) which was recorded and the intensity profile of the three fluorescent signals under the line of interest quantitated. The cells were then categorized according to the β-cat:E-cad intensity ratios of the membrane line of interest (Fig. 3G) and tabulated (Fig. 3I).

### Statistics

Analyses were conducted using Microsoft Excel’s data analysis plugin and GraphPad Prism’s statistical analysis tools. Ordinary one way ANOVA were used to compare different categories, time-points, perturbations as well as regions of the crypts. Correlation analysis (computing of Pearson’s correlation coefficient) was applied to the group of cells isolated based on the membrane β-catenin/E-cadherin expression levels. Significance and confidence level testing was based on a 95% confidence interval.

## Additional Information

**How to cite this article**: Tan, C. W. *et al*. Analysis of Wnt signalling dynamics during colon crypt development in 3D culture. *Sci. Rep*. **5**, 11036; doi: 10.1038/srep11036 (2015).

## Supplementary Material

Supplementary Information

Supplementary Video S1

Supplementary Video S2

Supplementary Video S3
