# Supplementary material for: Analysis of Wnt signalling dynamics during colon crypt development in 3D culture
Source: Sci Rep. 2015 Jun 18;5:11036. doi: 10.1038/srep11036 (PMC4471889; doi:10.1038/srep11036)
Supplement: Supplementary Information [file srep11036-s1.pdf]

SUPPLEMENTARY INFORMATION

Analysis of Wnt signalling dynamics during colon crypt development in 3D culture

Chin Wee Tan[1, 2,*], Yumiko Hirokawa[1] & Antony W. Burgess[1,2,3]

[1] Structural Biology Division, The Walter and Eliza Hall Institute of Medical Research, 1G Royal Parade, Parkville, VIC 3052 Australia;

[2] Department of Medical Biology, University of Melbourne, 1G Royal Parade, Parkville, VIC 3052 Australia;

[3] Department of Surgery, University of Melbourne, Royal Melbourne Hospital, Parkville, VIC 3050, Australia;

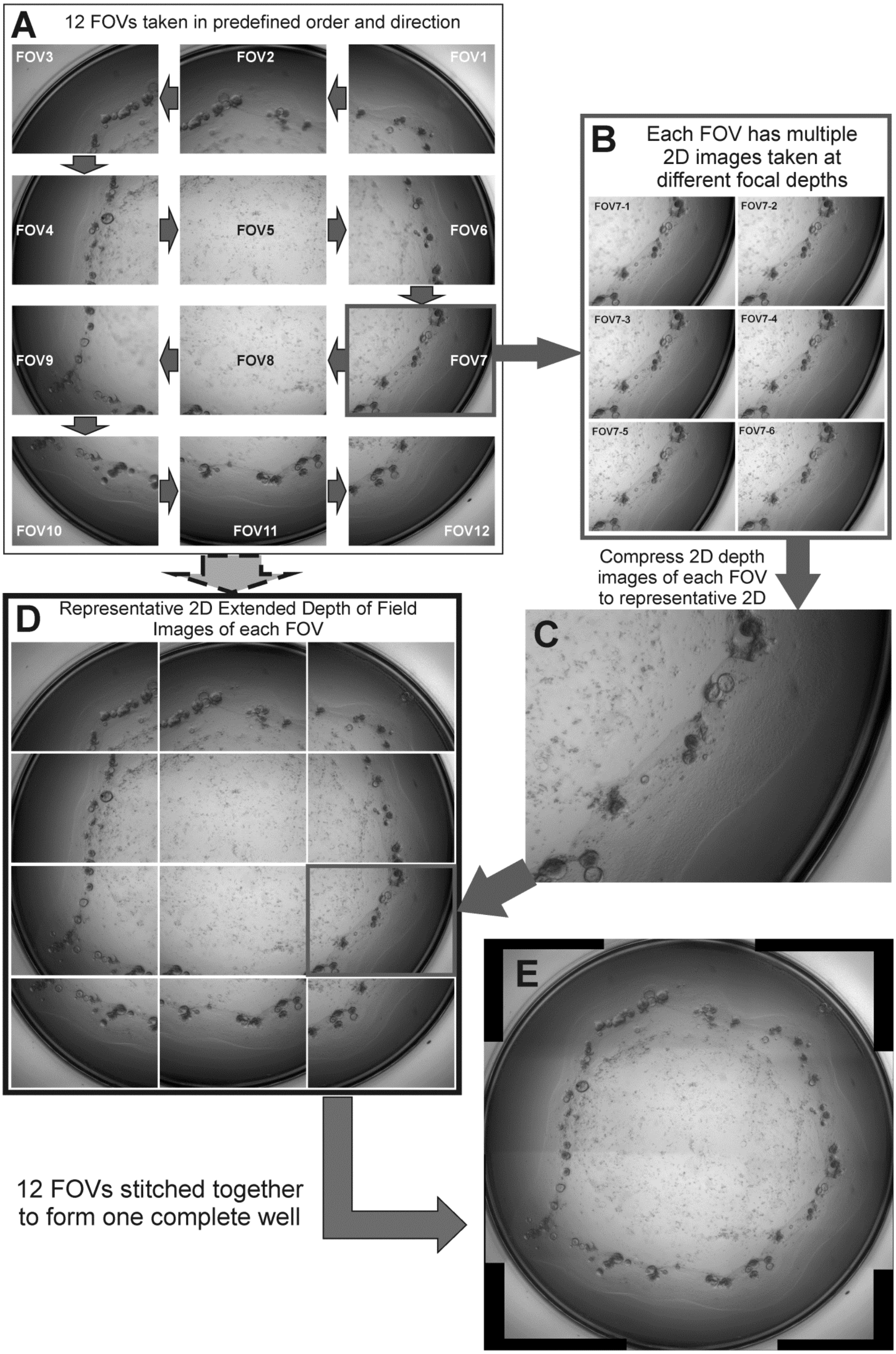
A
12 FOVs taken in predefined order and direction
FOV3
FOV2
FOV1
FOV4
FOV5
FOV6
FOV9
FOV8
FOV7
FOV10
FOV11
FOV12
B
Each FOV has multiple 2D images taken at different focal depths
FOV7-1
FOV7-2
FOV7-3
FOV7-4
FOV7-5
FOV7-6
Compress 2D depth images of each FOV to representative 2D
C
D
Representative 2D Extended Depth of Field Images of each FOV
E
12 FOVs stitched together to form one complete well

Supplementary Figure S1. Colon crypt culture microscopy and image processing setup. Bright field microscopy was conducted using a Nikon Eclipse Ti-U microscope with a 4x objective lens. Imaging was conducted daily, to acquire time-lapse images of crypt development. (A) For each well of the culture, 12 fields of views (FOVs) were defined and imaged in a pre-defined order (i.e. grid wise, snake by rows, from left then down). (B) Each FOV consist of multiple 2D images acquired at different depth/focus and organized into FOV-specific folders. Each folder contains a dataset specific to a culture well, position and time point. (C) The images in each FOV folder were organized into an image stack and compressed using the "Extended depth of field" plugin[37] into a 2D representative (2D-EDOF) image and renamed with the FOV number on the grid in panel A. (D) The twelve 2D-EDOF images were then processed using the "Grid/Collection Stitching" plugin to create one complete image of the culture well as shown in panel E. A series of stitched images were generated at different tiling overlap thresholds between FOVs from which the best reconstructed image was visually selected. This process (from compressing the images to the generation of the series of whole well images) has been automated using a Fiji/ImageJ script.

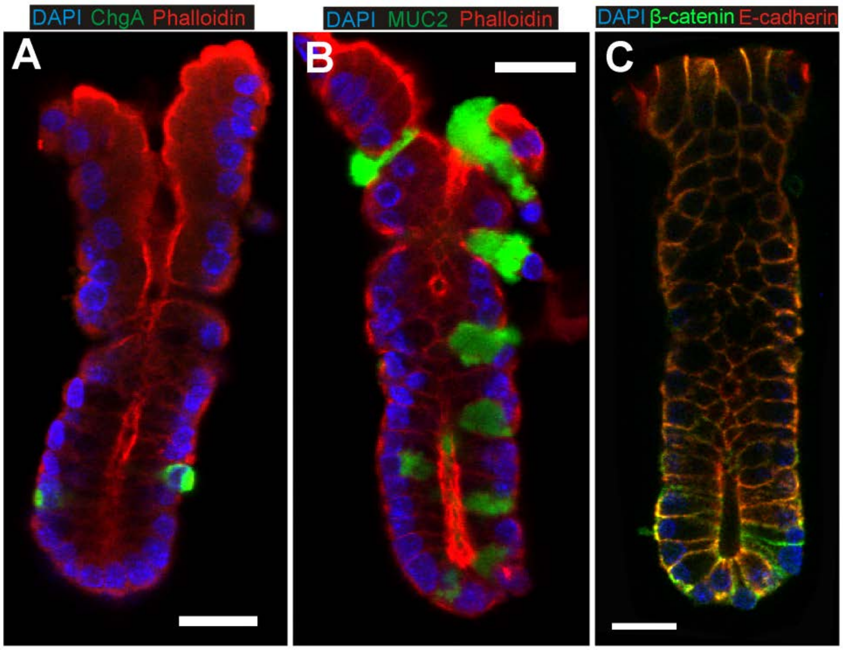

Supplementary Figure S2. Confocal immunofluorescence images of isolated colon crypts from wild-type C57BL/6 mouse stained for Chromogranin A, mucin 2, F-actin, β-cat and E-cad.

Confocal 2D images of crypts isolated from wildtype C57BL/6 mice, showing the expression and location of (A) chromogranin A and F-actin, (B) Muc2 and F-actin as well as (C) β-catenin and E-cadherin. DAPI was used to counter stain for the nucleus. Scale bar: 20μm

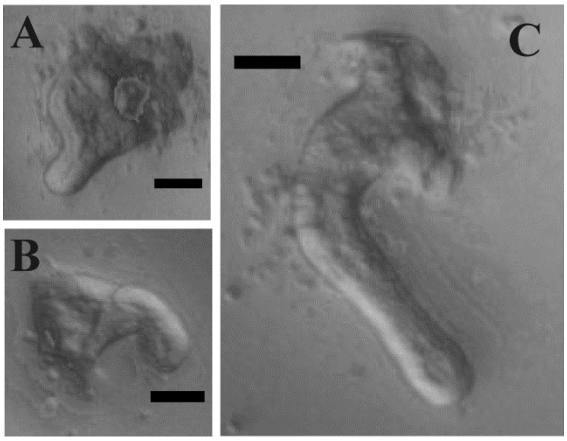

Supplementary Figure S3. Morphological differences in colon crypt developments between Tg(A33) mice and wild-type C57BL/6 mice.

2D-EDOF images of colon crypts grown in culture (using the setup described in Fig.1A) derived from (A) wildtype C57BL/6, Tg(A33-CreERT2);APC$^{fl/fl}$ (B) Cre-negative and (C) Cre-positive mice. Clear differences in crypt morphology were observed in the Cre-positive cultures with 15 out of 197 colonoids scored, developing new crypts having lengths greater than 160µm (greater than the average length of isolated crypts from distal colon of C57BL/6 mice, 154±21µm[26]). No such long crypts were observed in colonoids derived from either Cre-negative Tg(A33-CreERT2);APC$^{fl/fl}$ or wild-type C57BL/6 mice. Scale bar: 50µm

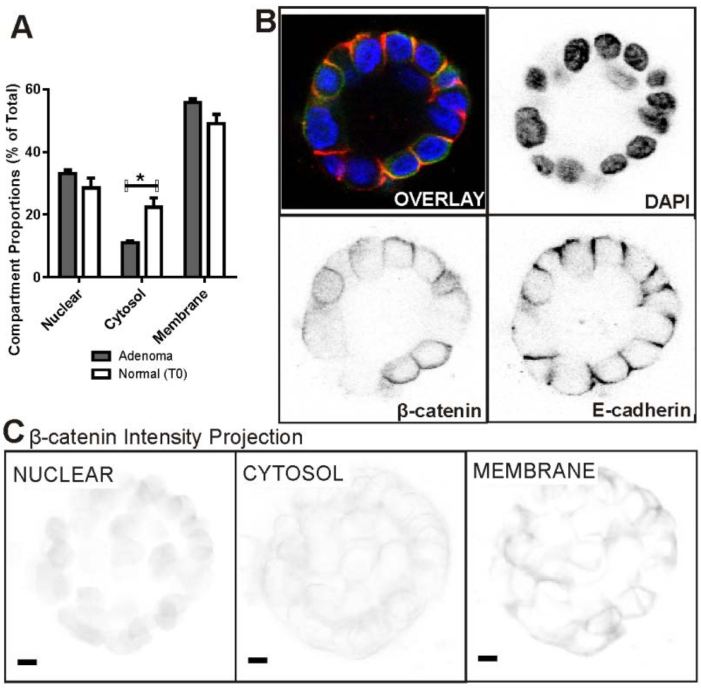

Supplementary Figure S4. Compartmental β-catenin ratio and localisation of adenoma colonospheres .

(A) Compartment β-catenin proportions of adenoma colonospheres as compared with that of colonospheres derived from normal C57 mice. A significant lower cytosolic β-catenin proportion (* $p<0.05$, ± SEM) was observed in the adenoma colonospheres compared to the normal. (B) 2D confocal section of an adenoma colonosphere showing the RGB overlay, DAPI, β-catenin and E-cadherin expression. Isolated β-catenin high cells can be observed amongst β-catenin low cells. (C) 2D intensity β-catenin projection images showing the nuclear, cytosolic and membrane localisation levels of β-catenin in the adenoma colonosphere. A low uniform expression was observed for cytosol β-catenin localisation while higher intensity patches can be seen in the nuclear and membrane β-catenin localisation projection images, indicating a β-catenin localisation towards the nuclear and membrane compartment in some cells of colon adenoma colonospheres. Statistical tests: single-factor anova. Scale bar: 5µm

Supplementary Video S1. 3D animation of a rotating day 10 crypt-shaped colonoid.

A multichannel 3D confocal image stack (DAPI in blue, β-catenin in green and E-cadherin in red) of a day 10 crypt-shaped colonoid derived from the colon culture with noggin withdrawn from day 2. The colonoid was rotated along the vertical axis of the image stack showing the lumen opening at the top and round crypt base at the bottom. Heterogeneous expression of β-catenin and E-cadherin can be seen along the length of the colonoid with patches of red, orange, green and yellow. Movie created using Fiji 3D Viewer[38].

Supplementary Video S2. 3D animation of a rotating day 3 crypt-shaped colonoid.

A multichannel 3D confocal image stack (DAPI in blue, β-catenin in green and E-cadherin in red) of a day 3 crypt-shaped colonoid derived from the colon culture with standard medium. The colonoid was rotated along the vertical axis of the image stack showing the lumen opening at the top and round crypt base at the bottom. Clusters of heterogeneous β-catenin and E-cadherin expression (highlighted by the patches of yellow, green and orange) near the crypt-base can be clearly seen. Movie created using Fiji 3D Viewer[38].

Supplementary Video S3. 3D animation of a rotating adenoma cyst.

A multichannel 3D confocal image stack (DAPI in blue, β-catenin in green and E-cadherin in red) of adenoma cyst derived from the colon adenoma culture of $APC^{min/+}$ mouse. The cyst was rotated along the vertical axis of the image stack showing the “bowl” shaped structure, particular the flattened bottom and thicken circumference wall. Movie created using Fiji 3D Viewer[38].
